# Domestically Acquired *Campylobacter* Infections in Finland

**DOI:** 10.3201/eid1001.020636

**Published:** 2004-01

**Authors:** Antti Vierikko, Marja-Liisa Hänninen, Anja Siitonen, Petri Ruutu, Hilpi Rautelin

**Affiliations:** * Haartman Institute Department of Bacteriology and Immunology, Helsinki, Finland; † Helsinki University Central Hospital Laboratory Diagnostics, Helsinki, Finland; ‡ University of Helsinki Department of Food and Environmental Hygiene, Helsinki, Finland; § National Public Health Institute, Helsinki, Finland

**Keywords:** *Campylobacter*, *Campylobacter jejuni*, Gastroenteritis, Serotype, Finland

## Abstract

*Campylobacter jejuni* isolates (n = 533) from domestic cases diagnosed in Finland during a 3-month peak period were studied. The highest rate was observed among those 70–74 years of age. Domestic *C. jejuni* isolates were especially frequent in the eastern districts. Six serotypes covered 61% of all *C. jejuni* isolates.

In developed countries, campylobacters are the most common culture-confirmed bacterial causes of gastroenteritis (*1*,*2*). The most common species identified in patients is *Campylobacter jejuni.* Since 1998, campylobacters also have been the most common bacterial enteropathogens detected in Finnish patients. A similar increasing trend has been recognized in many other European countries, such as Sweden, Denmark, and the United Kingdom (*3*–*5*). The epidemiology and modes of transmission are not well known, but risk factors for acquiring *Campylobacter* infection include handling or eating chicken, barbecuing, drinking unpasteurized milk or contaminated water, and traveling abroad (*1*,*6*–*8*).

 Some 30%-40% of *Campylobacter* infections detected in Swedish persons have been acquired in Sweden (*3*). Yet, in Denmark, approximately 80% of human infections are of domestic origin (*4*), suggesting that differences in risk factors may exist in these neighboring countries. In Finland, since 1994, all clinical microbiology laboratories are required to notify the National Infectious Disease Register (NIDR) of all *Campylobacter* findings based on culture, but no data on the distribution of domestically acquired and imported *Campylobacter* infections are collected.

We attempted to collect all *Campylobacter* isolates cultured in Finland from clinical stool samples of patients with domestically acquired infections during the seasonal peak of *Campylobacter* infections in 1999 and to analyze the heat-stable serotypes of *C. jejuni* strains.

## The Study

All clinical microbiology laboratories culturing campylobacters in Finland were asked to collect domestic *Campylobacter* isolates detected from human clinical fecal samples. Isolates collected on July 1 through September 30, 1999, from patients who had not been abroad for 2 weeks before becoming ill, were considered of domestic origin and were included in the study. Information on foreign travel, received by the physicians, was collected by the local clinical microbiology laboratories when culture results were reported to the clinical unit. The isolates were sent to Helsinki University Central Hospital (HUCH) Laboratory Diagnostics along with information about the patient (date of birth, sex, recent travel history) and the isolate (date of stool sampling, hippurate hydrolysis result). Consecutive isolates from the same patient were excluded. Isolates were stored at –70°C before serotyping. Hippurate-positive (*C. jejuni*) isolates were subsequently serotyped based on heat-stable Penner’s (Pen) antigens by passive hemagglutination using a serotyping set including 25 antisera (Campylobacter Antisera Seiken Set, Denka Seiken Co., Tokyo) as earlier described (*9*).

A total of 3,303 *Campylobacter* cases in Finland in 1999 were reported to NIDR; of these, 1,412 (43%) cases were diagnosed during our study period. In the present study, a total of 551 *Campylobacter* isolates were collected from patients who had presumably acquired their infections in Finland. The absence of case linkage between the two data sources prevents exact correlation between them; however, the number of domestic cases from the isolate collection is approximately 40% of the number of cases in NIDR for the same period. Of the strains collected, 533 (97%) were *C. jejuni* and 18 (3%) were *C. coli*. (Consecutive isolates related to outbreaks were not detected.)

**Figure 1:**
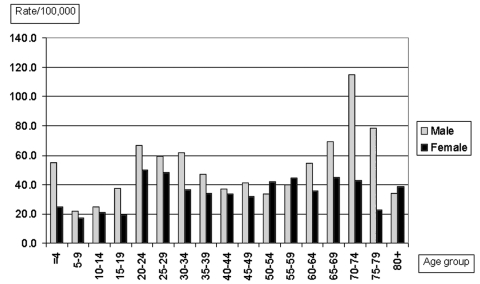
Domestically acquired *Campylobacter jejuni* infections in Finland by age and sex, July–September 1999.

The 533 case-samples of domestic *C. jejuni* infection collected within a population of 5.17 million yields a rate of 41.2 domestic *C. jejuni* cases per 100,000 inhabitants for the 3-month period. A higher proportion of patients were male (304 males, 57%). The rate of domestic *C. jejuni* infections by age group varied from 19.6 to 72.8 per 100,000 inhabitants for the 3-month period, with the highest rates observed among those >60 years old, among young adults (20–34 years of age), and among children <5 years of age (Figure 1).

**Figure 2:**
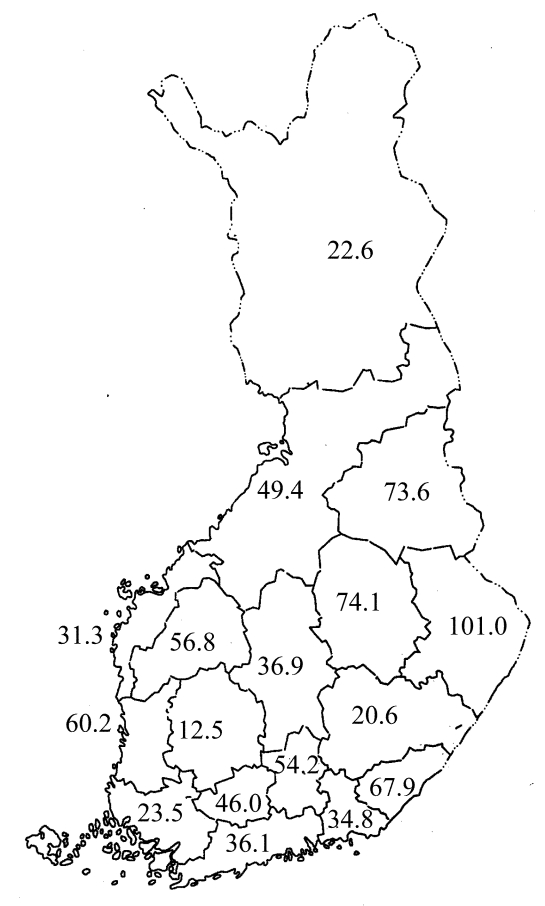
Rate of domestically acquired *Campylobacter jejuni* infections in Finland per 100,000 inhabitants, July–September 1999.

 Based on the municipality of the clinical unit from which the stool culture had been sent, several eastern hospital districts had high rates of domestic *Campylobacter* cases during the study period; the 95 % confidence intervals for these cases did not overlap those in several southern and western hospital districts with low rates (Figure 2). In some eastern districts, domestically acquired *Campylobacter* infections comprised even more than 80% of all cases reported to NIDR in July. In the other hospital districts, a peak was also demonstrated in July; then the number of domestic *C. jejuni* cases declined in August, and the number of isolates collected in September comprised only 10% of all strains isolated during the 3-month study period.

 The seasonal distribution of the different serotypes is presented in the Table. The predominant serotypes (Pen 1,44, Pen 2, Pen 4-cluster, Pen 6,7, Pen 12, and Pen 27) comprised 61% of the isolates (Table). No clear geographic differences in the distribution of serotypes were found. None of the strains reacted with the antisera against serotypes Pen 10, 32, 38, or 45. Reactions with serotype Pen 3 and Pen 8 antisera were only present in complex serotypes. Serotypes Pen 5, 11, 18, 19, 31, 52, and 55 (not included in the Table) were so uncommon that they together represented approximately 5% of all the isolates.

**Table:** Penner serotypes of *Campylobacter jejuni* isolates detected from human fecal samples of patients in Finland with domestically acquired infections, July–September 1999

Penner serotype	No. (%)^a^			
	July	August	September	3-month period
1,44	17 (5)	4 (3)	3 (6)	24 (5)
2	7 (2)	29 (21)	10 (19)	46 (9)
4-cluster^b^	34 (10)	19 (14)	6 (11)	59 (11)
6,7	75 (22)	11 (8)	1 (2)	87 (16)
12	32 (9)	23 (17)	16 (30)	71 (13)
15	4 (1)	1 (1)	1 (2)	6 (1)
21	8 (2)	0 (0)	2 (4)	10 (2)
23, 36, 53	2 (1)	2 (1)	3 (6)	7 (1)
27	30 (9)	9 (7)	0 (0)	39 (7)
37	2 (1)	7 (5)	0 (0)	9 (2)
41	6 (2)	0 (0)	0 (0)	6 (1)
57	16 (5)	3 (2)	0 (0)	19 (4)
Untypeable	36 (11)	19 (14)	5 (9)	60 (11)
Complex	64 (19)	7 (5)	4 (7)	75 (14)
Total	342	137	54	533

^a^Uncommon serotypes Pen 5, 11, 18, 19, 31, 52, and 55 not included. ^b^4-cluster, serotypes 4, 13, 16, 43, 50.

**Figure 3:**
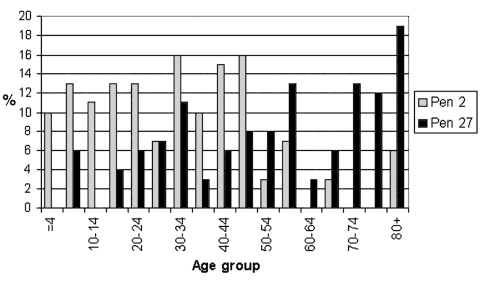
Pen 2 and Pen 27 serotypes among domestically acquired *Campylobacter jejuni* infections in Finland by age, July–September 1999.

 The mean age of all case-patients was 41.4 years. Patients infected with *C. jejuni* serotype Pen 15 (mean age 23.5 years, 95% CI 6.0, 41.0) or serotype Pen 2 (mean age 32.7 years, 95% CI 26.3, 39.0) were significantly younger than those infected with serotype Pen 27 (mean age 49.6 years, 95% CI 42.7, 56.4) or serotype Pen 6,7 (mean age 46.8 years, 95% CI 42.2, 51.4). The age distribution of serotypes Pen 2 and Pen 27 is shown in Figure 3. Differences between the age distributions of case-patients infected with other serotypes were not significant.

## Conclusions

All hospital districts in Finland participated in this study, and the rate of domestically acquired *C. jejuni* infections, as measured by the number of isolates sent, was highest in several eastern hospital districts. The higher rates cannot be explained by urban versus rural lifestyle alone since the western districts in the study included rural and urbanized areas in an approximately similar proportion as in the eastern districts.

The six most commonly found serotypes accounted for approximately 60% of the cases. We have studied human domestic *Campylobacter* infections in Finland since 1995 (*9*–*11*) and found the same serotypes to be common among *C. jejuni* isolates collected during July through September, although annual variation in the relative proportion of predominant serotypes is evident. Serotypes Pen 1,44, Pen 2, and Pen 4-cluster have been found relatively often in human fecal samples in Denmark (*4*,*12*), England (*13*), and New Zealand (*14*), but serotypes Pen 6,7, Pen 12, and Pen 27 have been less common (<3% each) in those studies.

From May to September 1999, a total of 1,132 chicken flocks, representing most of the chicken meat produced and consumed in Finland, were monitored for campylobacters. Thirty-one *C. jejuni*–positive flocks were detected; the most common Penner serotypes identified were Pen 6,7 and Pen 12, found mainly from July through August (*15*). These particular serotypes were also commonly found in human infections in the present study in July and in August. Serotypes Pen 27 and Pen 4-cluster were also found in the chickens (*15*). These results indicate some overlapping between human and chicken strains at the serotype level. Humans and chicken may share a common source for *C. jejuni,* or humans may acquire the infection from contaminated chicken meat.

The highest rate of domestic *C. jejuni* infection was found among patients in the age group of 70–74 years, accompanied by an above average rate among patients 65–69 years old. This is the first report of such an age peak for *C. jejuni* (*1*,*3*,*4*). Furthermore, the age distribution of patients infected with isolates of certain serotypes suggests that older people in Finland may have somewhat different sources of infection than younger people. Serotypes Pen 6,7 and Pen 27, in particular, appeared to be relatively common among the elderly patients. In our study, serotype Pen 2 was more common than Pen 27 in all age groups <50 years of age (excluding the age group 25–29 years) but less common in all older groups. Since serotypes Pen 6,7 and Pen 27 are also common in chicken (*15*), there may be a link between raw poultry handling or chicken consumption practices and *C. jejuni* in the elderly.

 During the 1999 seasonal peak, domestically acquired cases of *C. jejuni* in Finland were caused by the same serotypes that are most commonly found in other developed countries, yet some common serotypes in our study seem to be less common elsewhere. High rates were observed among elderly men and in the east of Finland. The six most common serotypes covered 61% of all isolates. We recommend further study to determine if the results were specific to the time period or representative of a persistent phenomenon.
